# Changes in Metal Solubility in PM_2.5_ in Xi’an City Under Clean Heating Policies: Effects of Emission Source and Aerosol Acidity

**DOI:** 10.3390/toxics14020168

**Published:** 2026-02-12

**Authors:** Hongyu Yan, Pingping Liu, Yuhao Dong, Chuchen Li, Zhiwei Xue, Jing Xue, Jian Sun, Hongmei Xu

**Affiliations:** 1 Department of Environmental Science and Engineering, Xi’an Jiaotong University, Xi’an 710049, China; yhyhy0318@163.com (H.Y.); dongyh0221@163.com (Y.D.); sunjian0306@mail.xjtu.edu.cn (J.S.); 2 No. 11 Oil Production Plant, Changqing Oilfiled Company, PetroChina, Qingyang 745100, China; lcc2_cq@petrochina.com.cn; 3 No. 203 Research Institute of Nuclear Industry, Xi’an 710086, China; xuezhiwei2026@163.com; 4 Key Laboratory for Space Bioscience and Biotechnology, School of Life Science and Technology, Northwestern Polytechnical University, Xi’an 710129, China; xuejing0089@nwpu.edu.cn

**Keywords:** clean heating, PM_2.5_, metal solubility, acidity, source contribution

## Abstract

Clean heating policies were implemented in rural areas of Shaanxi Province in 2017 to alleviate severe air pollution. To evaluate their impacts on bioavailability of PM_2.5_-bound metals, the influence of emission sources and aerosol acidity on PM_2.5_-bound metal solubility was explored in Xi’an over three policy-defined periods between 2016 and 2021. Results showed that aerosol pH increased progressively from 4.81 ± 1.82 to 5.29 ± 1.79 following policy implementation, closely associated with reductions in SO_2_ and NO_2_ concentrations due to emission controls. Metal concentrations decreased significantly over the study period. In contrast, metal solubility exhibited clear source-dependent variations. Solubilities of metals associated with coal combustion, biomass burning, and industrial activities (As, Cd, Pb, K and Zn) decreased by 16.6–50.5% with weakening aerosol acidity. In contrast, solubilities of metals related to vehicle exhaust, oil fuel combustion and dust (Cu, V, Ni, Ti and Fe) increased by 38.3–56.8%, indicating enhanced influence of emission processes. Source apportionment demonstrated that mixed contributions of coal combustion, biomass burning and industrial activities to total and water-soluble metals decreased by 12% and 11.2%, respectively, while contribution from secondary atmospheric processes increased by 4% and 3.8%. These findings highlight that clean heating policies reshape both metal sources and atmospheric chemical environments, thereby altering metal dissolution characteristics and bioavailability.

## 1. Introduction

Although metals in PM_2.5_ (aerosols with an aerodynamic diameter ≤ 2.5 µm) are generally present at trace levels, they exert a significant influence on the toxicity of airborne aerosols [1]. Metals exist in both water-insoluble and water-soluble forms in aerosols. Compared with their water-insoluble forms, water-soluble metals exhibit higher bioavailability and thus pose greater adverse effects on human health [2,3]. Previous studies have demonstrated that certain water-soluble transition metals (Fe, Cu, and Mn) can enhance the generation of reactive oxygen species (ROS) in the human body. This process triggers oxidative stress and inflammatory responses, thereby contributing to the onset of both chronic and acute diseases [4,5]. In addition, even at relatively low concentrations, water-soluble toxic metals (As, Cd, Cr and Pb) have been associated with elevated carcinogenic and non-carcinogenic risks in both adults and children [6].

The concentrations of water-soluble metals or metal solubility are influenced by aerosol acidity [7]. More acidic conditions facilitate metal solubilization and enhance their bioaccessibility [8]. Meteorological factors also play a role in metal solubility. For example, relative humidity alters aerosol liquid water content, thereby influencing metal dissolution [9]. Notably, aerosol acidity is controlled by its water-soluble ionic composition as well as ambient relative humidity and temperature [10]. In particular, sulfate and nitrate are formed through atmospheric chemical reactions of acidic gases (SO_2_ and NO_x_) emitted from coal combustion and biomass burning [11,12,13]. The ionic balance between these ions and the alkaline component, ammonium, collectively determines the proton activity in aerosols [14]. When the total molar equivalent of sulfate and nitrate exceeds the neutralizing capacity of ammonium, excess protons lead to a decrease in the particulate-phase pH, resulting in the acidic environment [15]. The acidic environment inhibits the hydrolysis and precipitation of metal ions, allowing them to exist as free hydrated ions or soluble complexes. Organic ligands, such as oxalate, can also regulate metal solubility through complexation [16]. These processes collectively alter the potential toxicity of metals.

In addition to aerosol acidity, metal solubility is also closely related to emission sources [17]. Metals emitted from different sources exhibit substantial differences in chemical speciation and particle size distribution, leading to pronounced source-dependent dissolution behavior. Anthropogenic metals such as Cu, Zn, As and Pb produced by combustion processes are generally enriched in fine particles and exhibit high chemical reactivity, resulting in relatively high solubility, which often exceeds 40%. In contrast, crustal metals such as Fe, Al and Ti originating from soil dust are mainly embedded in stable mineral matrices with low reactivity, and their solubility is typically lower than 20% [17,18,19,20]. In recent years, northern China has implemented a series of clean heating policies to mitigate air pollution [21,22,23,24]. Studies have shown that replacing traditional residential coal combustion with cleaner heating options, such as natural gas heating, electric heating, and solar-based heating systems, has substantially reduced the contribution of residential coal combustion, thereby lowering emissions of coal-related metals (As, Cd, Pb and Zn) and consequently reducing emissions of acidic precursors such as SO_2_ and NO_x_ [25]. This reduction weakens the formation of sulfates and nitrates in the atmosphere, thereby decreasing aerosol acidity. Such changes may limit the conversion of certain transition metals (Fe, Cu and Mn) from insoluble to soluble forms under acidic conditions [21], ultimately altering the solubility patterns and toxicity of particulate metals [26]. As coal-related sources have diminished, the relative contributions of vehicle exhaust, industrial emissions, and road dust have increased, leading to a more complex composition and solubility characteristics of metals in PM_2.5_ [27]. These findings suggest that clean heating policies can exert complex, dual effects on metal solubility through modifications in both emission source structure and aerosol acidity, yet their impacts warrant systematic evaluation at the regional scale.

Xi’an, the core city of northwestern China, has long been affected by serious air pollution due to high concentrations of PM_2.5_ [28]. To address this issue, clean heating policies were implemented in Xi’an in 2017, including coal-to-gas and coal-to-electricity transitions for residential heating, which were associated with substantial reductions in ambient air pollutant concentrations [29]. However, the impacts of these policies on emission source structure, aerosol acidity and consequently on metal solubility remain insufficiently understood. Previous studies have largely focused on total metal concentrations and associated health risks, whereas limited attention has been given to metal solubility [30,31,32]. This study involved the collection of PM_2.5_ samples during a five-year interval from June 2016 to May 2021. Total metals, water-soluble metals, and water-soluble inorganic ions were quantified. Based on these datasets, we investigated temporal variations in metal solubility and explored the key factors influencing these changes. This work provides a scientific foundation for the assessment of clean heating policy performance in Xi’an, while also contributing to the refinement of future air pollution control measures.

## 2. Materials and Methods

### 2.1. Sample Collection

Located in southeastern central Xi’an, the sampling site experienced substantial air pollution due to emissions from adjacent residential areas and nearby arterial roads. The sampling instruments were installed on the roof of a 15 m tall building within Xi’an Jiaotong University. The exact location has been reported in detail in our previous studies [33,34,35,36]. Three temporal sampling phases were established in this study to reflect the rollout stages of clean heating policies in Xi’an city. According to policy implementation status, the sampling intervals were defined as Y1 (June 2016–May 2017), Y2 (June 2018–May 2019), and Y3 (June 2020–May 2021), representing the pre-, mid-, and post-policy periods, respectively. PM_2.5_ sampling was carried out with a high-volume sampler (HVS-PM_2.5_, Tisch Environmental Inc., Cleves, OH, USA), yielding a total of 256 samples. Specifically, the numbers of valid annual samples for Y1 (June 2016–May 2017), Y2 (June 2018–May 2019) and Y3 (June 2020–May 2021) were 66, 96 and 94, respectively. During the heating seasons of these three periods, 23, 25 and 23 valid samples were obtained, respectively, whereas during the non-heating seasons, the corresponding numbers were 43, 71 and 71. Information on sampler flow rate configuration and PM_2.5_ mass concentration measurements is available in prior work [28]. All collected filters were immediately packaged in pre-baked aluminum foil, kept in contamination-free storage boxes, and maintained at −18 °C before analysis.

### 2.2. Chemical Analysis

A total of twelve metal elements (As, Cd, Cr, Cu, Fe, K, Ti, V, Mn, Zn, Ni, and Pb) were analyzed using ICP-OES (ICPE-9000, Shimadzu, Kyoto, Japan). The detailed analytical procedures have been reported [3]. Briefly, one-quarter of each filter (4.34 cm^2^) was sectioned into small fragments and transferred into a Teflon digestion vessel for acid treatment. A mixed acid solution consisting of 4.5 mL HNO_3_, 1.5 mL HCl and 1 mL HF was added to each sample. Samples were first pre-digested on a hot plate at 150 °C for 20 min. Microwave digestion was subsequently performed using a Multiwave Go system (Anton Paar, Graz, Austria). The digestion procedure consisted of two stages. The temperature was first increased stepwise to 120, 160, and 185 °C within 50 min. Subsequently, the digest was evaporated on a hot plate at 180 °C until the volume reached about 0.5 mL. After digestion, the solutions were brought to 10 mL with 2% HNO_3_ and passed through a 0.22 μm PES membrane filter. A blank vessel was prepared in parallel for each batch to evaluate analytical accuracy.

The concentrations of water-soluble metals were analyzed using an ICP-MS system (NexION 350D, PerkinElmer, Shelton, CT, USA). The detailed analytical procedures are as follows. Half of the filter sample (8.68 cm^2^) was finely cut and extracted using 7 mL of ultrapure water with a resistivity of 18.3 MΩ. The extraction process involved an initial 1 h ultrasonic bath, followed by 2 h of shaking on an orbital shaker (30 rpm), and a final 10 min settling period. The extract was then filtered through a 0.22 μm polyethersulfone (PES) membrane filter. During batch testing, standard solutions were inserted for verification every 15 samples. If the relative error exceeded 10%, the standard curve was redrawn. Before processing a new batch, several samples analyzed in the earlier batch were randomly rechecked to evaluate instrumental performance.

The concentrations of water-soluble inorganic ions were measured by ion chromatography (Dionex Integrion HPIC, Thermo Fisher Scientific Inc., Waltham, MA, USA). The concentrations of five cations (Na^+^, NH_4_^+^, K^+^, Mg^2+^, and Ca^2+^) were measured with a CS12A column, employing 20 mM methanesulfonic acid as the mobile phase at a flow rate of 1 mL min^−1^. The four anions (F^−^, Cl^−^, NO_3_^−^, and SO_4_^2−^) were analyzed using AS11-HC and AG11-HC columns. Further information is available in prior publications [37,38].

All tubes and polyethylene containers were thoroughly cleaned prior to use. The procedure included soaking in 20% HNO_3_ for 24 h, five rinses with deionized water, 30 min of ultrasonic cleaning, another five rinses, and drying at 60 °C. Laboratory blanks were subjected to identical analytical procedures to ensure measurement accuracy. Blank subtraction for all measured metal species and inorganic ions was conducted based on field blank filters.

### 2.3. Data Analysis

#### 2.3.1. Sulfur Oxidation Ratio (SOR), Nitrogen Oxidation Ratio (NOR) and Neutralization Ratio

SOR and NOR serve as indicators of the extent to which sulfur and nitrogen undergo secondary conversion processes. The degree of sulfate and nitrate neutralization by ammonia is commonly characterized by the neutralization ratio, a parameter frequently used to infer aerosol acidity. The corresponding values were derived according to the equations given below [39,40]:

(1) $$SOR=\frac{n({SO_{4}}^{2-})}{n(SO_{2})+n({SO_{4}}^{2-})}$$

(2) $$NOR=\frac{n({NO_{3}}^{-})}{n(NO_{2})+n({NO_{3}}^{-})}$$

(3) $$R_{\mathrm{neutral}}=\frac{\left[{NH_{4}}^{+}\right]}{2[{SO_{4}}^{2-}]+[{NO_{3}}^{-}]}$$

where n(SO_4_^2−^) and n(SO_2_) represent the respective concentrations of SO_4_^2−^ and SO_2_. n(NO_3_^−^) and n(NO_2_) represent the concentrations of NO_3_^−^ and NO_2_, respectively. All parameters are expressed in mol m^−3^.

#### 2.3.2. Aerosol Acidity

The thermodynamic equilibrium composition of aerosols was simulated using the ISORROPIA-II model in the forward mode under metastable assumptions. These selected configurations have been widely applied to ensure the accuracy of aerosol pH predictions. Model inputs included Na^+^, NH_4_^+^, K^+^, Mg^2+^, Ca^2+^, Cl^−^, NO_3_^−^, SO_4_^2−^, NH_3_, HCl, HNO_3_, ambient temperature (T) and relative humidity (RH). Meteorological variables, including temperature (T) and relative humidity (RH), were obtained from daily records provided by the China Meteorological Administration http://www.weather.com.cn/ (accessed on 10 July 2025).

In this study, gaseous precursors (NH_3_(g), HNO_3_(g), and HCl(g)) were not directly measured on site. Instead, we followed the iterative approach proposed in previous studies [8,16,41,42] to estimate aerosol pH and aerosol liquid water content (ALWC). Briefly, the first model run assumed that the sum of measured aerosol species and estimated gas-phase species (HNO_3_, HCl and NH_3_) constituted the total input. This step produced the first set of gas-aerosol partitioning outputs. For the second run, the gaseous outputs from the initial run were added back to the original aerosol dataset and treated again as the total input, similar to the first iteration. The procedure was repeated until the change of NO_3_^−^ outputs was less than 1% of the total mass, which was considered the convergence threshold. The iterative calculation process can be expressed as follows:

(4) $$\mathrm{Input}{\left[C_{Aerosol}+C_{Gas}\right]}_{N+1}=C_{Aerosol}+{\left[C_{Gas}\right]}_{N}$$

(5) $$L=\left|\frac{{\left[C_{{NO_{3}}^{-}}\right]}_{N+1}-{\left[C_{{NO_{3}}^{-}}\right]}_{N}}{{\left[C_{{NO_{3}}^{-}}\right]}_{N}}\right|\times 100\%$$

In this formulation, *C_Aerosol_* is the observed concentration of NO_3_^−^ (or NH_4_^+^, Cl^−^), and *C_Gas_* is the concentration of the associated gaseous species HNO_3_(g) (or NH_3_(g), HCl(g)). The term [*C_Gas_*]*_N_* represents the gas-phase concentration simulated by ISORROPIA-II during the Nth run (*N* ≥ 1). The iterative process was stopped once *L* < 1%.

Based on ISORROPIA-II outputs, aerosol *pH* was derived from aqueous H^+^ levels and corresponding aerosol liquid water content, as defined in Equation (6):

(6) $$pH=-\log_{10}\frac{1000\times {H^{+}}_{\left(aq\right)}}{ALWC}$$

#### 2.3.3. Source Apportionment

Source apportionment was conducted using the EPA PMF 5.0 model to explore the origins of metal solubility, considering twelve metals and their water-soluble components (As, Cd, Cr, Cu, Fe, K, Ti, V, Mn, Zn, Ni, and Pb). The input data for the total metal analysis included PM_2.5_ mass concentration, total concentrations of twelve metals, concentrations of three water-soluble ions (SO_4_^2−^, NO_3_^−^ and NH_4_^+^), three gaseous pollutants (SO_2_, NO_2_ and CO) and their associated uncertainties. The input data for the water-soluble metal analysis included PM_2.5_ mass concentration, total concentrations of twelve metals, concentrations of three water-soluble ions (SO_4_^2−^, NO_3_^−^, and NH_4_^+^) and their associated uncertainties. The PMF model framework is defined by Equation (7) [43] and the uncertainties were calculated according to Equations (8) and (9) [44]:

(7) $$X_{ij}=\sum_{k=1}^{p}g_{ik}f_{kj}+e_{ij}$$

where the summation term (Σ*k* = 1 to *p*) represents the combined contribution of all identified source factors, *p* is the total number of factors resolved by the PMF model, *i* and *j* denote the *ith* sample and the *jth* species, respectively, *x_ij_* represents the concentration of species *j* in sample *i*, *g_ik_* and *f_kj_* represent the contribution of factor *k* to sample *i* and the species profile of factor *k*, respectively, and *e_ij_* denotes the residual matrix, with *g_ik_* and *f_kj_* constrained to be non-negative. The measured concentration is expressed as a linear combination of the contributions from all resolved sources plus the residual term.

When concentrations are lower than the *MDLs*:

(8) $$\mathrm{Uncertainty}=\frac{5}{6}\times MDL$$

If concentrations are higher than the *MDLs*:

(9) $$\mathrm{Uncertainty}=\sqrt{(\mathrm{EF}\times {\mathrm{concentration})}^{2}+{\left(0.5\times MDL\right)}^{2}}$$

The uncertainty calculations in Equations (8) and (9) follow the standard procedures recommended in the U.S. EPA PMF 5.0 user guide [45]. *MDL* denotes the method detection limit of the analytical instrument for each element and *EF* represents the percentage uncertainty of the measured concentrations. In this study, *EF* was set to 10%.

PMF model runs evaluated factor numbers varying from 3 to 7. Based on interpretable factor profiles reported in previous studies, four optimal factors were selected for both total metals and water-soluble metals. The optimal solution was identified by minimizing the Q value, achieving a Q_Robust_/Q_Theo_ ratio of less than 2, and ensuring that most standardized residuals fell within the ±3 range.

## 3. Results and Discussion

### 3.1. Changes in Aerosol Acidity Under Clean Heating Policies

**Figure 1:**
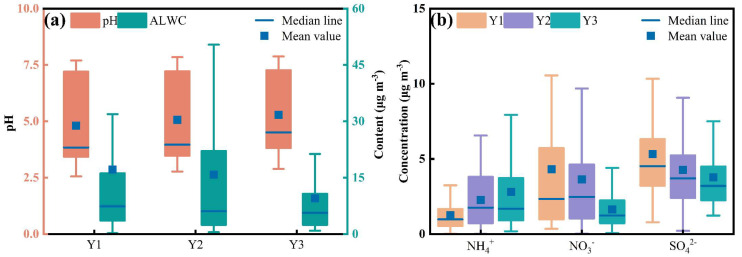
Changes in annual average concentrations of different substances: (**a**) aerosol acidity and liquid water content; (**b**) ammonium, nitrate, and sulfate. Y1, Y2, and Y3 correspond to the pre-policy (2016–2017), mid-policy (2018–2019), and post-policy (2020–2021) periods, respectively.

The study first investigated the changes in aerosol acidity before, during, and after the implementation of clean heating policies. As shown in Figure 1, the annual average aerosol pH increased during the policy implementation period, with values of 4.81 ± 1.82, 5.07 ± 1.88 and 5.29 ± 1.79 for the pre-, mid- and post-implementation phases, respectively. The annual average aerosol liquid water content decreased across the three phases, with values of 17.10 ± 5.26, 15.82 ± 3.06, and 9.56 ± 2.81 μg m^−3^, respectively. Consistent with previous studies [46], an increasing trend in aerosol pH was also observed during the COVID-19 period. This period was characterized by lockdown-related reductions in industrial activity and energy demand, which resulted in a short-term decrease in coal consumption and were accompanied by lower SO_2_ and NO_2_ concentrations [47]. According to Table S1, annual mean SO_2_ and NO_2_ concentrations exhibited decreases of 60.4% and 30.8%, respectively, across the three periods. These changes were more pronounced during the heating season (Figure S1). Compared with Y1, Y3 exhibited a 21.8% increase in aerosol pH and a 55.2% decrease in aerosol liquid water content, accompanied by reductions of 69.2% in SO_2_ and 40.5% in NO_2_. The emissions of acidic gases (SO_2_ and NO_2_) primarily originated from coal combustion [48]. Winter represented the peak period of coal use, and the implementation of coal-to-gas and coal-to-electricity transitions for residential heating substantially reduced coal consumption during the heating season [21]. According to the Xi’an Statistical Yearbook (Figure S1), coal consumption decreased by 64.3% from 2016 to 2020, which directly contributed to the reductions in SO_2_ and NO_2_ emissions. Although the sulfur oxidation ratio (SOR) and nitrogen oxidation ratio (NOR) (Table 1) increased from Y1 to Y2, indicating enhanced atmospheric oxidation under reduced primary emissions, their stabilization from Y2 to Y3 suggests that the availability of precursors (SO_2_ and NO_2_) became increasingly limited.

In addition, solar radiation plays an important role in regulating the photochemical environment and thereby influences the secondary transformation of SO_2_ and NO_2_. Ozone (O_3_), used as an indicator of photochemical activity driven by solar radiation, shows (Table S4) significant associations with the sulfur oxidation ratio (SOR) and nitrogen oxidation ratio (NOR), suggesting that the oxidation efficiencies of SO_2_ and NO_2_ were maintained or even enhanced under photochemically active conditions [49]. Although aerosol liquid water content decreased overall during the study period, the increasing sensitivity of secondary formation processes to humidity implies a synergistic effect between photochemical oxidation and humidity-dependent heterogeneous or aqueous-phase reactions.

The reductions in SO_2_ and NO_2_ concentrations indirectly suppressed the formation of water-soluble sulfate and nitrate in PM_2.5_. After entering the atmosphere, SO_2_ and NO_2_ were oxidized by hydroxyl radicals (·OH) to form sulfuric and nitric acids, which rapidly dissociated into H^+^, sulfate and nitrate in the particle phase [14]. Compared with Y1, Y3 exhibited decreases of 29.0%, 61.8% and 78.3% in the annual average concentrations of SO_4_^2−^, NO_3_^−^, and H^+^ (Table S3), respectively, while the annual average concentration of NH_4_^+^ increased by 124.8%. Based on the calculated neutralization index, R_neutral_ increased by 18.1% in Y3 relative to Y1 (Table 1), indicating an enhanced alkaline environment. In addition to compositional effects, meteorological conditions contribute significantly to changes in pH [7,50]. Relative humidity decreased markedly in Y3 compared with Y1 (Table S2). This reduction limited aerosol water uptake, leading to lower aerosol liquid water content and suppressed the partitioning of gaseous HNO_3_ and HCl into the particle phase. In addition, reduced aerosol water hindered the dissociation of HSO_4_^−^. Collectively, these results drove a year-by-year increase in aerosol pH and a concurrent decrease in aerosol liquid water content.

### 3.2. The Effects of Aerosol Acidity on Metal Solubility

As shown in Tables S5 and S6, changes over time in total and water-soluble concentrations of twelve metals (Fe, Ti, Mn, Cu, Pb, Cd, As, K, Zn, Cr, Ni, and V) were observed across the three periods (Y1–Y3) in Xi’an. The annual average mass concentrations of total metals were 14,010 ± 3035.4, 9711.6 ± 3596.6, and 5955.6 ± 2490.4 ng m^−3^, accounting for 9.5%, 9.3% and 8.1% of the average PM_2.5_ mass concentration, respectively (Table S1). The annual average mass concentrations of water-soluble metals were 800.4 ± 518.4, 540.1 ± 229.2 and 383.4 ± 171.8 ng m^−3^, accounting for 5.7%, 5.6%, and 6.4% of the corresponding total metal mass concentrations, respectively. Both exhibited declining trends over time. Higher total metal levels were generally observed during heating periods, whereas water-soluble components contributed less and showed pronounced variability. The observed behavior can be attributed to the combined impacts of source emission processes and subsequent atmospheric aging on metal solubility in particles.

To further investigate this effect, we analyzed the variations in source-specific metal solubility in relation to aerosol acidity during the policy implementation periods. Characteristic metals for coal combustion sources include As, Cd, and Pb [51,52], while K serves as a marker for biomass burning [53]. The coal-to-gas/electricity conversion policy primarily targets the reduction in residential coal and biomass combustion. In contrast, Cu is widely adopted as a key indicator for vehicular emissions [54]. Ti and Fe, which are largely derived from mineral components of soil and characterized by minimal solubility, serve as robust indicators of natural dust inputs [55,56]. Fuel oil combustion is characterized by elevated Ni and V, reflecting their high abundance in residual oils derived from diesel, fuel oil, coal combustion, and high-temperature metallurgical processes [57,58]. Additionally, industrial activities release complex mixtures of metals, including impurities originating from ore materials. Emissions from non-ferrous metal smelting are characterized by elevated levels of Zn, Cr, and Mn, making these elements representative of industrial activities [59,60].

**Figure 2:**
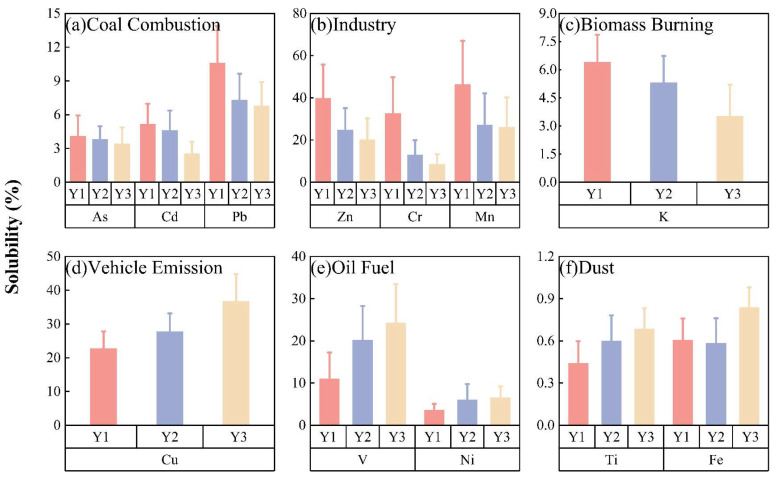
Characteristics of interannual variability in source-dependent metal solubility.

As shown in Figure 2, the solubility of multiple metals exhibited pronounced interannual variations from Y1 (pre-policy period) to Y3 (post-policy period), broadly consistent with the observed increasing trend in aerosol pH. The solubility of metals associated with coal combustion, biomass burning, and industrial sources generally decreased, with As, Cd, Pb, K and Zn declining by 16.6%, 50.5%, 35.9%, 45.0% and 49.3%, respectively, from Y1 to Y3. In contrast, the solubility of traffic-related metals increased markedly over the same period, with Cu, V and Ni rising by 38.3%, 54.5% and 45.8%, respectively. Seasonal analysis further showed (Figure S2) that, during the pre-policy period, the solubility of most metals was markedly higher in heating seasons than in non-heating seasons, with combustion-related metals exhibiting heating-seasons solubility that was 5–10% higher than that in non-heating seasons. However, this seasonal contrast weakened progressively during Y2 and Y3, indicating that the influence of wintertime acidic conditions on metal dissolution was substantially mitigated following the implementation of clean heating measures.

The specific response of metal solubility to aerosol acidity reflects differences in dissolution pathways driven by chemical environment evolution. In the early phase of policy implementation (Y1), elevated proton concentrations and higher liquid water content promoted proton-promoted dissolution, enhancing the release of embedded metals (particularly As, Pb and K) from combustion-derived particles. As the aerosol pH increased from 4.81 ± 1.82 in Y1 to 5.29 ± 1.79 in Y3, coupled with a decrease in ALWC from 17.10 ± 5.26 μg m^−3^ to 9.56 ± 2.81 μg m^−3^, the efficiency of proton-promoted dissolution declined, leading to reduced solubility of these metals. In contrast, traffic-related metals such as Cu, V and Ni, which are primarily associated with finer particles and more labile chemical forms, exhibited increased solubility despite the rise in aerosol acidity. This suggests that factors such as particle size, surface reactivity, and heterogeneous processing may partly decouple their dissolution behavior from the bulk aerosol pH.

### 3.3. The Role of Atmospheric Chemical Processes in Metal Solubility

Previous studies [61] have shown that acidic species in condensed atmospheric aerosols can promote iron dissolution through sulfate processing during atmospheric aging. Iron-rich particles from industrial emissions become coated with acidic sulfates formed from the condensation of sulfuric acid produced by SO_2_ oxidation, facilitating the dissolution of otherwise insoluble iron. This acid-driven mechanism, which requires aerosol pH to overcome particle buffering capacity, may also apply to other metals. Similar processes are likely relevant for nitrate, as nitric acid formation can likewise enhance aerosol acidity and promote metal dissolution.

**Figure 3:**
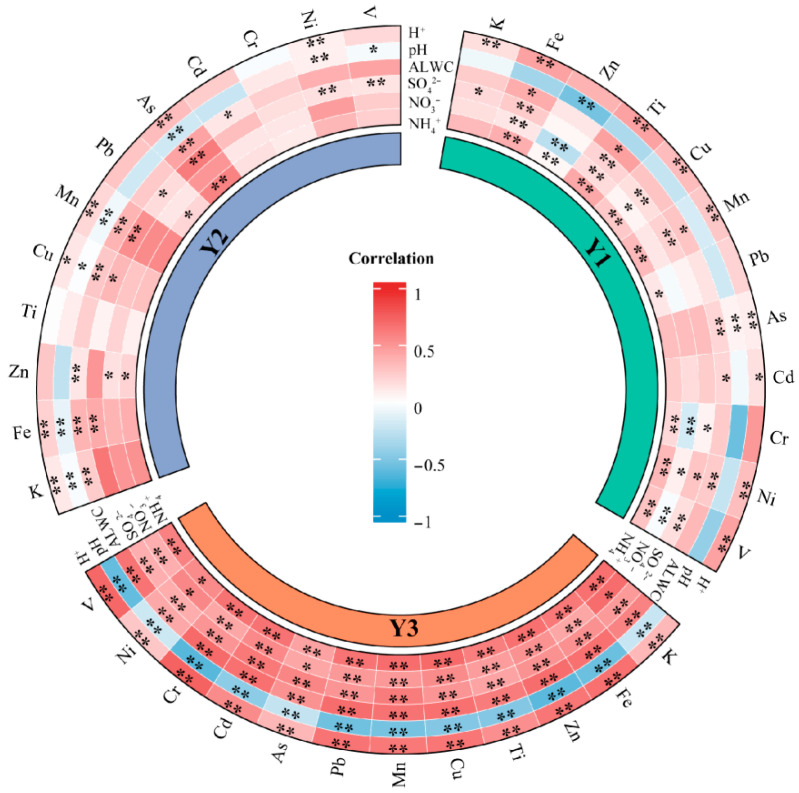
The spearman’s correlation between metal solubility and H^+^, pH, ALWC, sulfate, nitrate, ammonium (* for *p* < 0.05, ** for *p* < 0.01).

Spearman correlation analysis (Figure 3) was applied to examine the relationships between the solubility of twelve metals and H^+^, pH, aerosol liquid water content (ALWC), sulfate, nitrate and ammonium. The specific correlation coefficients are provided in Supplementary Table S7. Relationships between metal solubility and acidity related parameters exhibited clear source-dependent characteristics and systematic evolution from Y1 to Y3. In Y1 (pre-policy period), metals primarily associated with coal combustion, biomass burning, and industrial emissions (e.g., As, Cd, Pb, K and Zn) showed strong correlations with bulk aerosol acidity indicators, including negative associations with pH and positive associations with proton concentration (H^+^). These patterns indicate that proton promoted dissolution dominated the mobilization of combustion-related metals under highly acidic conditions when primary emissions were intensive [8].

As clean heating policies were implemented and coal related emissions declined (Y2 and Y3), the correlation structure shifted markedly. For combustion and industry related metals, correlations with pH and H^+^ weakened progressively, while associations with sulfate and nitrate also became less pronounced, reflecting a reduced direct influence of source driven acidity on metal dissolution. In contrast, correlations between the solubility of several metals (e.g., Fe, Mn and Zn) and aerosol liquid water content (ALWC) strengthened, suggesting an increasing role of aqueous phase processes and atmospheric aging in regulating metal solubility under less acidic but more processed aerosol conditions [62]. Traffic and heavy oil combustion related metals (Cu, V and Ni) exhibited the most distinct transition. From Y1 to Y3, their solubility showed progressively stronger correlations with ALWC and secondary inorganic components, whereas associations with bulk acidity indicators remained weak. This pattern indicates that the dissolution of these metals became increasingly governed by atmospheric environmental conditions rather than by direct emission characteristics. Overall, the strengthened correlations observed from Y1 to Y3 reflect a transition from source dominated controls on metal solubility toward a regime increasingly regulated by secondary atmospheric processes following the implementation of clean heating policies.

### 3.4. Explanatory of Pollution Sources for Metal Solubility Decreases

**Figure 4:**
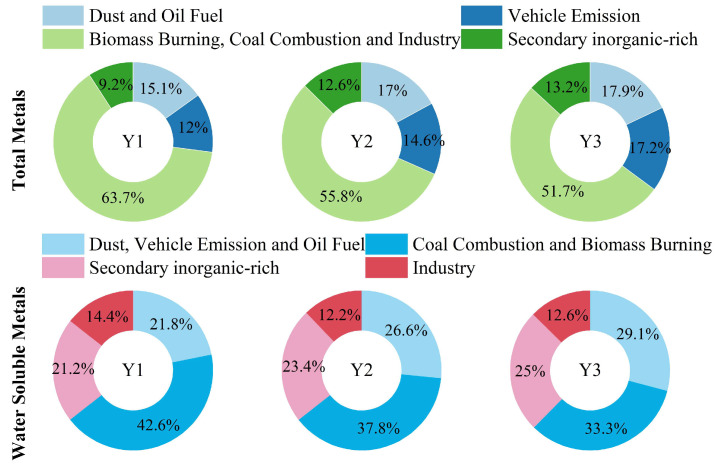
Source contributions of total metals and water-soluble metals.

To further elucidate the role of emission sources in regulating metal solubility, the EPA PMF 5.0 model was applied separately to total metals and water-soluble metals (Figures S3 and S4), and the source contributions were directly compared (Figure 4). Clear shifts in source contributions were observed across the three policy phases, reflecting the restructuring of emission sources induced by clean heating measures. Prior to policy implementation (Y1), biomass burning, coal combustion, and industrial sources dominated total metal concentrations, accounting for 63.7% of the total metal mass, with a comparable contribution (57.0%) to water-soluble metals. This consistency indicates that combustion- and industry-related emissions were not only the primary sources of particulate metals but also major contributors to their soluble fractions under strongly acidic conditions [63].

Following the implementation of clean heating policies, the contributions from biomass burning, coal combustion, and industrial sources declined substantially, decreasing to 51.7% for total metals and 45.8% for water-soluble metals in the Y3 (post-policy period). The more pronounced reduction in their contribution to water-soluble metals than to total metals suggests that source reduction alone cannot fully explain the observed changes in metal solubility. Instead, the concurrent weakening of aerosol acidity, as discussed in Section 3.1, likely suppressed proton promoted dissolution of metals associated with these sources, thereby disproportionately reducing their soluble fractions. This interpretation is consistent with the declining solubility of combustion- and industry-related metals (As, Cd, Pb, K and Zn) observed from Y1 to Y3.

In contrast, the relative contributions of vehicle emission, oil fuel combustion, dust and secondary processes increased for both total and water-soluble metals during the policy periods. Notably, these sources exhibited a larger increase in their contributions to water-soluble metals than to total metals, highlighting their enhanced efficiency in generating soluble metal fractions under evolving atmospheric conditions. Traffic-related and oil-combustion-related sources, characterized by finer particle sizes and more labile metal forms (e.g., Cu, V and Ni), appear particularly sensitive to atmospheric processing. As aerosol acidity weakened, aqueous-phase processes and aerosol liquid water availability became increasingly important in sustaining metal solubility, as demonstrated by the strengthened correlations between these metals and ALWC in Section 3.3. Meanwhile, the increasing contribution of secondary processes further underscores the growing importance of atmospheric chemical aging in regulating metal dissolution.

**Figure 5:**
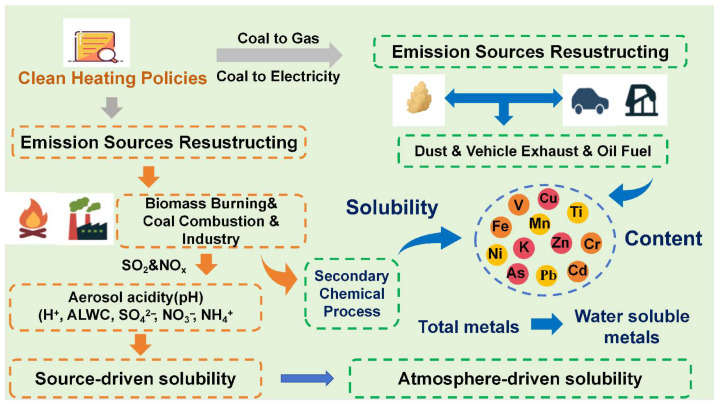
The impact pathway of Clean heating policies on metal solubility (The color of arrows in the picture has no representative meaning).

In summary, clean heating policies influence metal solubility through a dual mechanism (Figure 5). On one hand, the reduction in combustion-related emissions directly decreases the input of highly soluble metals. On the other hand, the concurrent weakening of aerosol acidity alters dissolution pathways, shifting the dominant control of metal solubility from source-driven acidity effects toward atmospheric process driven regulation.

## 4. Conclusions

This study demonstrates that clean heating policies in Xi’an have reshaped PM_2.5_ metal solubility through coupled changes in emission sources and aerosol chemical conditions, rather than through reductions in particulate mass alone. The transition away from coal combustion systematically weakened aerosol acidity and modified aqueous phase environments, resulting in decreased solubility of metals associated with residential combustion and industrial sources, while enhancing the solubility of traffic-related metals under less acidic yet chemically active conditions. Correlation analysis further reveals a shift in the dominant controls on metal dissolution from proton-driven processes to a multi-factor regime regulated by aerosol acidity, liquid water content and secondary inorganic components. These results indicate that clean energy transitions can reorganize the bioavailable fraction of particulate metals in a source and metal-specific manner. Future studies should explicitly link acidity-mediated metal dissolution with oxidative potential and toxicological endpoints to better elucidate how policy-driven changes in aerosol chemistry translate into particle toxicity and human health impacts, thereby supporting the development of more health-oriented air pollution control strategies.

## Figures and Tables

**Table 1:** Changes in SOR, NOR and R_neutral_ across the three periods.

Index		SOR	NOR	R_neutral_
Annual average	Y1	0.18	0.02	0.61
	Y2	0.33	0.07	0.72
	Y3	0.34	0.07	0.77
Heating seasons	Y1	0.14	0.03	0.79
	Y2	0.30	0.10	0.85
	Y3	0.32	0.08	0.89
Non-heating seasons	Y1	0.20	0.02	0.51
	Y2	0.35	0.06	0.72
	Y3	0.34	0.07	0.68

## Data Availability

The original contributions presented in this study are included in the article/Supplementary Materials. Further inquiries can be directed to the corresponding authors.

## Supplementary Materials

The following supporting information can be downloaded at: https://www.mdpi.com/article/10.3390/toxics14020168/s1, Figure S1: Changes in Coal and Natural Gas Consumption and Motor Vehicle Ownership in Xi’an City from 2016 to 2021; Figure S2: Seasonal variation characteristics of source-dependent metal component solubility; Figure S3: Source profiles of Total Metals; Figure S4: Source profiles of Water-soluble Metals; Table S1: Changes in pollutant concentrations during three periods (the values are presented in mean ± standard deviation); Table S2: Annual average changes in temperature and relative humidity over three periods; Table S3: Changes in sulfate concentration during different periods (the values are presented in mean ± standard deviation); Table S4: Spearman correlation analysis between sulfur oxidation rate (SOR), nitrogen oxidation rate (NOR), temperature (Temp), relative humidity (RH), and ozone (* for *p* < 0.05, ** for *p* < 0.01); Table S5: Changes in total metal concentration during different periods (the values are presented in mean ± standard deviation); Table S6: Changes in water-soluble metal concentration during different periods (the values are presented in mean ± standard deviation); Table S7: Spearman correlation coefficients between metal solubility and H^+^, pH, ALWC, sulfate, nitrate, and ammonium in the different periods (* for *p* < 0.05, ** for *p* < 0.01).
