# Supplementary material for: Changes in Metal Solubility in PM2.5 in Xi’an City Under Clean Heating Policies: Effects of Emission Source and Aerosol Acidity
Source: Toxics. 2026 Feb 12;14(2):168. doi: 10.3390/toxics14020168 (PMC12945187; doi:10.3390/toxics14020168)
Supplement: Supplementary file 1 [file toxics-14-00168-s001.zip › toxics-4137448-supplementary.pdf]

Article

Changes in Metal Solubility in $PM_{2.5}$ in Xi'an City under Clean Heating Policies: Effects of Emission Source and Aerosol Acidity

Hongyu Yan [1], Pingping Liu [1,*], Yuhao Dong [1], Chuchen Li [2], Zhiwei Xue [3], Jing Xue [4], Jian Sun [1] and Hongmei Xu [1,*]

[1] Department of Environmental Science and Engineering, Xi'an Jiaotong University, Xi'an 710049, China; yhyhy0318@163.com (H.Y.); dongyh0221@163.com (Y.D.); sunjian0306@mail.xjtu.edu.cn (J.S.)
[2] No.11 Oil Production Plant, Changqing Oilfiled Company, PetroChina, Qing yang, 745100, China; lcc2_cq@petrochina.com.cn
[3] No.203 Research Institute of Nuclear Industry, Xi'an, 710086, China; xuezhiwei2026@163.com
[4] Key Laboratory for Space Bioscience and Biotechnology, School of Life Science and Technology, Northwestern Polytechnical University, Xi'an 710129, Shaanxi, China; xuejing0089@nwpu.edu.cn
* Correspondence: liupingping@xjtu.edu.cn (P.L.); xuhongmei@xjtu.edu.cn (H.X.)

Table S1. Changes in pollutant concentrations during three periods(the values are presented in mean ± standard deviation).

Pollutant (μg m^{-3})		$PM_{2.5}$	SO_2	NO_2	CO	O_3
Annual	Y1	102.12±81.61	20.32±12.36	62.15±23.10	1.69±0.90	79.01±47.52
	Y2	63.28±38.57	10.81±5.71	50.88±18.28	1.00±0.35	87.53±52.07
	Y3	44.57±28.74	8.04±2.89	43.00±14.34	0.73±0.24	94.02±58.27
Heating season	Y1	189.17±122.77	34.35±9.94	80.57±23.63	2.53±0.97	44.12±31.78
	Y2	91.52±44.98	16.41±7.38	64.56±19.20	1.30±0.40	42.34±16.80
	Y3	57.09±25.28	10.57±3.09	47.96±13.49	0.90±0.27	45.08±24.53
Non-heating season	Y1	55.56±40.91	12.81±4.31	52.30±15.59	1.29±0.40	90.87±48.23
	Y2	38.73±23.04	8.68±2.88	45.68±15.05	0.89±0.24	110.23±48.09
	Y3	40.52±28.78	7.23±2.31	41.39±14.32	0.68±0.20	113.04±54.75

Table S2. Annual average changes in temperature and relative humidity over three periods.

Meteorological conditions	Sampling periods		
	Y1	Y2	Y3
Temperature (℃)	16.75	16.01	15.35
Relative humidity (%)	62.89	59.13	57.26
Atmospheric pressure (pa)	1017.72	1016.39	1017.94
Wind speed (m s^{-1})	2.25	2.36	2.24

Table S3. Changes in sulfate concentration during different periods(the values are presented in mean ± standard deviation).

Concentration ($\mu g\ m^{-3}$)		H^+	pH	ALWC	Sulfate	Nitrate	Ammonium
Annual	Y1	$6.49\times10^{-6}\pm1.20\times10^{-5}$	4.82±1.82	17.19±5.26	5.33±3.30	4.32±2.76	1.26±1.13
	Y2	$5.74\times10^{-6}\pm1.10\times10^{-5}$	5.07±1.88	15.82±3.06	4.27±2.32	3.64±2.02	2.27±2.04
	Y3	$1.41\times10^{-6}\pm2.80\times10^{-6}$	5.29±1.79	9.56±2.81	3.79±2.24	1.65±0.40	2.81±2.68
Heating season	Y1	$6.77\times10^{-6}\pm1.69\times10^{-6}$	4.68±1.23	28.32±11.96	6.90±4.41	4.90±2.58	3.58±3.33
	Y2	$2.28\times10^{-6}\pm4.06\times10^{-6}$	5.11±1.69	14.20±5.41	4.91±2.07	4.50±2.52	2.94±1.87
	Y3	$1.30\times10^{-6}\pm1.95\times10^{-6}$	5.70±1.89	12.70±3.87	5.44±2.75	1.84±0.57	1.23±1.31
Non-heating season	Y1	$1.47\times10^{-6}\pm3.15\times10^{-6}$	5.61±1.94	7.89±2.63	2.90±1.23	1.01±0.73	0.74±0.53
	Y2	$7.80\times10^{-6}\pm1.32\times10^{-6}$	4.70±1.87	16.33±6.40	4.73±2.56	3.37±2.08	2.15±2.03
	Y3	$5.35\times10^{-6}\pm7.86\times10^{-6}$	4.87±1.84	12.96±3.83	4.06±2.37	3.18±1.98	2.05±2.06

Table S4. Spearman correlation analysis between sulfur oxidation rate (SOR), nitrogen oxidation rate (NOR), temperature (Temp), relative humidity (RH), and ozone(* for P<0.05, ** for P<0.01).

	**TEMP**			**RH**			**O_3**			**SOR**			**NOR**		
	Y1	Y2	Y3	Y1	Y2	Y3	Y1	Y2	Y3	Y1	Y2	Y3	Y1	Y2	Y3
TEMP	--	--	--												
RH	-.287*	0.123	0.118	--	--	--									
O_3	.785**	.850**	.791**	-.580**	-0.031	-.217*	--	--	--						
SOR	.394**	0.158	0.165	-0.062	.265*	.672**	0.021	.321**	.482**	--	--	--			
NOR	-.567**	-.640**	-.492**	0.141	.243*	.504**	.368**	.512**	.596**	.290*	.312**	.440**	--	--	--

Table S5. Changes in total metal concentration during different periods(the values are presented in mean ± standard deviation).

Metal/ng·m⁻³	Annual			Heating season			Non-heating season		
	Y1	Y2	Y3	Y1	Y2	Y3	Y1	Y2	Y3
As	130.26±606.52	93.01±51.63	71.98±19.77	90.88±5.73	71.12±9.73	57.69±14.60	94.46±66.89	158.17±712.93	72.33±22.62
Cd	17.00±12.27	23.75±103.51	14.08±4.32	22.26±7.02	14.08±4.32	12.29±3.43	13.43±13.74	28.15±121.71	14.06±4.88
Cr	25.87±25.45	38.38±162.37	17.44±7.43	21.32±4.86	16.84±4.87	17.57±5.40	28.95±32.46	46.66±190.73	17.38±8.13
Cu	169.95±308.07	138.99±77.63	175.58±121.93	181.90±389.58	146.89±80.04	144.17±56.24	161.85±240.98	135.95±77.00	188.14±138.18
Fe	4398.01±4782.74	3327.14±5637.25	3336.06±2959.63	3399.85±1241.18	2618.57±1824.78	3113.54±1502.80	5074.18±6036.40	3599.66±6529.54	3425.07±3375.99
K	6901.49±4269.28	4601.82±7349.19	2217.26±1593.31	7869.16±1786.00	4947.96±1488.10	2139.19±928.99	6245.98±5248.90	4468.69±8611.34	2248.48±1796.15
Mn	150.63±174.13	130.20±142.61	128.23±99.78	117.28±40.03	98.95±50.58	96.97±39.44	173.22±220.99	142.22±163.61	140.74±113.26
Ni	14.90±10.43	13.12±33.20	15.47±7.23	11.84±2.52	10.67±4.75	15.51±3.04	16.98±12.98	14.07±38.98	15.46±8.35
Pb	77.75±52.90	47.13±37.86	33.97±19.32	102.32±41.41	76.65±51.55	41.33±17.52	61.11±53.62	35.78±22.83	31.02±19.33
Ti	245.29±371.41	222.71±251.60	199.88±226.59	180.45±88.32	201.91±182.14	148.81±102.45	289.21±472.04	230.70±274.29	220.31±258.00
V	6.00±10.08	5.10±7.38	4.14±5.74	4.24±2.37	4.37±5.11	3.84±3.18	7.19±12.82	5.38±8.10	4.26±6.50
Zn	1224.71±1196.15	998.74±1610.23	142.81±65.21	2008.80±1118.42	1518.80±713.10	149.42±44.95	693.55±930.09	798.71±1807.10	140.17±71.83
∑Ms	14010.11±3035.47	9711.69±3596.62	5955.66±2490.41	13324.61±2389.38	9677.33±14534.55	6356.90±4873.17	12860.12±12104.56	9664.15±16985.85	6517.42±5547.04

Table S6. Changes in water-soluble metal concentration during different periods(the values are presented in mean ± standard deviation).

Metal/ng·m^{-3}	Annual average			Heating season			Non-heating season		
	Y1	Y2	Y3	Y1	Y2	Y3	Y1	Y2	Y3
As	3.07±1.42	3.06±5.61	2.48±1.77	3.89±1.74	3.47±1.90	2.40±1.11	2.51±0.78	2.90±6.50	2.51±1.98
Cd	1.18±0.87	0.64±1.26	0.35±0.30	1.77±0.98	0.97±1.02	0.44±0.31	0.78±0.50	0.52±1.33	0.31±0.29
Cr	6.85±3.48	6.41±38.66	1.59±3.22	7.44±3.93	1.85±0.72	2.55±5.84	6.45±3.10	8.16±45.45	1.19±0.62
Cu	74.51±211.81	42.34±50.90	66.59±59.74	107.52±328.38	40.14±23.86	66.92±52.04	52.14±44.92	43.19±58.16	66.45±62.98
Fe	63.79±41.78	11.92±10.68	19.82±52.59	79.00±52.93	16.38±10.60	34.22±95.36	53.49±28.24	10.21±10.27	13.90±11.57
K	226.67±133.73	201.68±191.68	130.43±89.87	339.02±127.16	325.29±144.72	184.58±97.00	150.56±68.93	154.14±186.88	108.18±77.00
Mn	53.50±27.21	26.39±21.70	30.84±24.21	69.76±29.52	32.38±12.29	26.17±12.83	42.49±18.95	24.09±24.04	32.76±27.41
Ni	0.85±0.33	0.59±0.82	0.51±0.23	0.94±0.34	0.65±0.22	0.62±0.21	0.79±0.31	0.57±0.96	0.47±0.23
Pb	20.83±17.20	5.19±9.47	2.74±4.89	31.01±21.74	10.44±14.93	4.15±7.84	13.93±7.89	3.17±5.08	2.16±2.82
Ti	0.73±0.40	0.75±0.68	0.81±0.55	0.80±0.32	1.17±0.60	0.99±0.60	0.69±0.44	0.59±0.64	0.73±0.51
V	1.04±0.52	0.69±1.36	0.51±0.26	1.24±0.50	0.63±0.22	0.61±0.21	0.91±0.49	0.72±1.59	0.47±0.27
Zn	107.82±89.29	67.66±80.67	50.85±47.41	158.05±83.13	106.71±67.44	59.72±45.70	73.86±76.02	53.34±80.96	47.21±47.92
∑WS-MS	800.45±518.43	540.19±229.25	383.42±171.89	859.81±410.30	567.34±359.57	407.51±174.10	398.61±179.05	300.92±378.28	276.34±224.16

Table S7. Spearman correlation coefficients between metal solubility and H^+, pH, ALWC, sulfate, nitrate, and ammonium in the different periods (* for P<0.05, ** for P<0.01).

Species	H^+			pH			ALWC			SO_4^{2-}			NO_3^-			NH_4^+		
	Y1	Y2	Y3	Y1	Y2	Y3	Y1	Y2	Y3	Y1	Y2	Y3	Y1	Y2	Y3	Y1	Y2	Y3
K	0.11	0.156	0.335**	-0.023	-0.068	-0.229*	0.237	0.238*	0.589**	0.655**	0.184	0.479**	0.499**	0.164	0.686**	0.520**	0.326**	0.679**
Fe	0.222	0.449**	0.676**	-0.098	-0.381**	-0.568**	0.360**	0.400**	0.683**	0.405**	0.248*	0.528**	0.355**	0.118	0.433**	0.385**	0.387**	0.636**
Zn	0.266*	0.370**	0.654**	-0.267*	-0.539**	-0.630**	0.12	0.123	0.687**	0.497**	0.035	0.515**	0.147	-0.288**	0.518**	0.212	0.043	0.677**
Ti	-0.004	0.447**	0.544**	0.092	-0.338**	-0.502**	0.233	0.456**	0.618**	0.061	0.211*	0.442**	0.213	0.175	0.466**	0.077	0.419**	0.608**
Cu	0.123	0.316**	0.645**	-0.009	-0.244*	-0.498**	0.271*	0.285**	0.685**	0.354**	0.159	0.513**	0.277*	0.05	0.546**	0.301*	0.262**	0.702**
Mn	0.19	0.271**	0.640**	-0.064	-0.171	-0.540**	0.357**	0.278**	0.670**	0.538**	0.218*	0.623**	0.553**	0.115	0.489**	0.555**	0.332**	0.695**
Pb	0.277*	0.205*	0.662**	-0.195	-0.197	-0.564**	0.259*	0.137	0.686**	0.171	0.06	0.506**	0.123	-0.035	0.487**	0.203	0.117	0.663**
As	0.321**	0.085	0.349**	-0.198	0.059	-0.250*	0.437**	0.198	0.522**	0.622**	0.316**	0.583**	0.499**	0.320**	0.429**	0.589**	0.308**	0.449**
Cd	0.238	0.147	0.547**	-0.231	-0.048	-0.461**	0.12	0.199	0.675**	0.311*	0.241*	0.639**	0.124	0.172	0.561**	0.145	0.241*	0.670**
Cr	-0.022	0.487**	0.740**	0.11	-0.565**	-0.644**	0.231	0.251*	0.703**	0.162	0.064	0.507**	0.117	-0.185	0.433**	0.103	0.183	0.673**
Ni	0.079	0.309**	0.269*	0.082	-0.243*	-0.210*	0.379**	0.249*	0.417**	0.148	0.280**	0.396**	0.467**	0.079	0.572**	0.348**	0.335**	0.546**
V	0.183	0.454**	0.712**	-0.043	-0.425**	-0.603**	0.437**	0.340**	0.663**	0.133	0.158	0.389**	0.245*	-0.016	0.390**	0.234	0.286**	0.603**

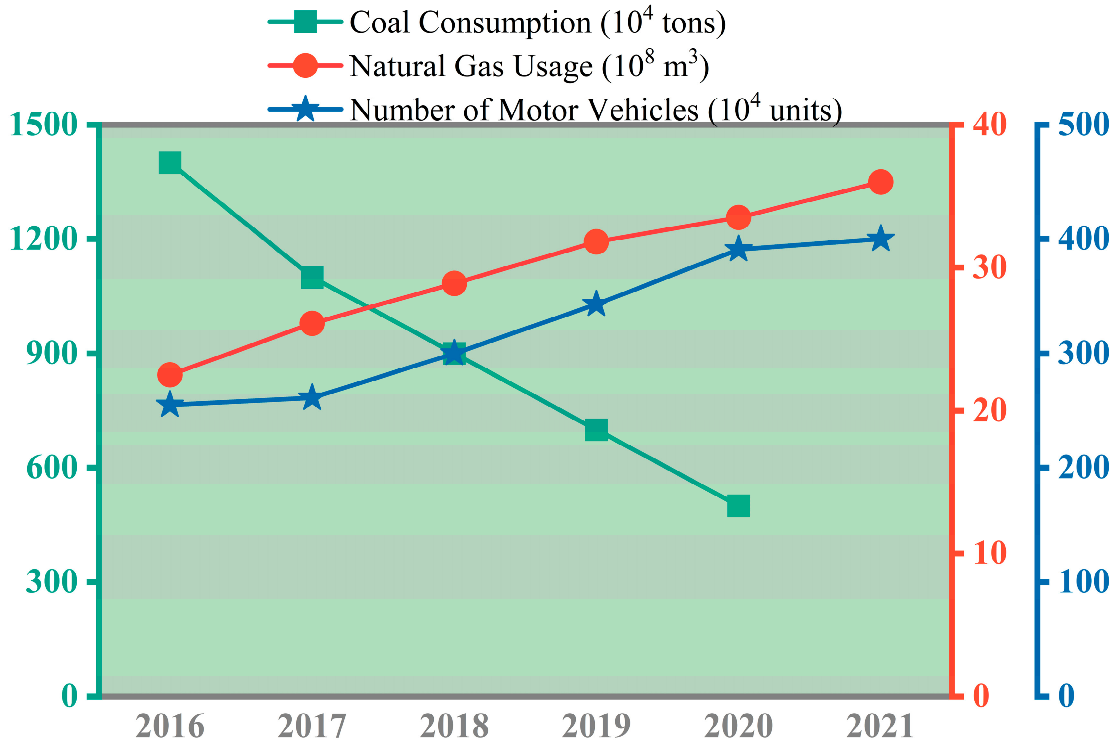

Figure S1. Changes in Coal and Natural Gas Consumption and Motor Vehicle Ownership in Xi'an City from 2016 to 2021.

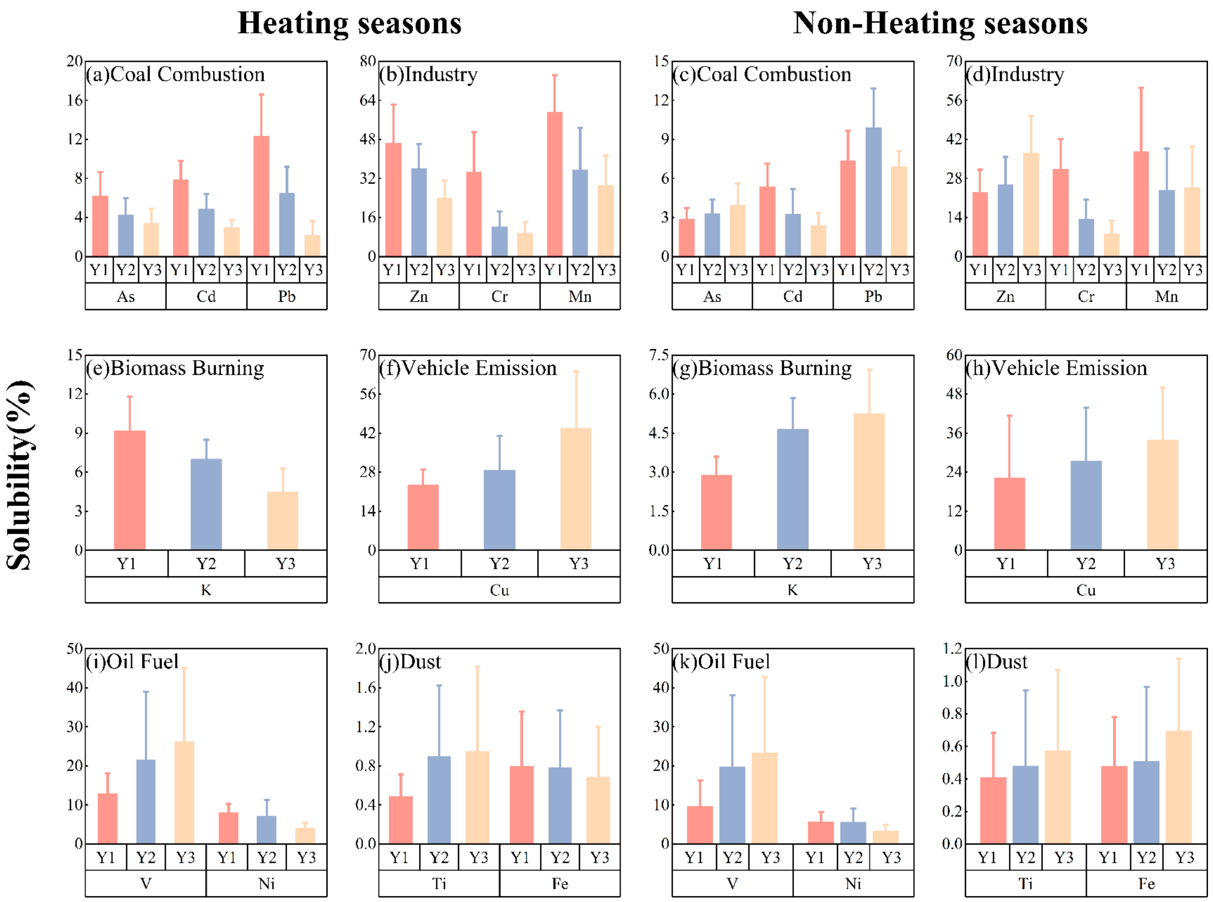

Figure S2. Seasonal variation characteristics of source-dependent metal component solubility.

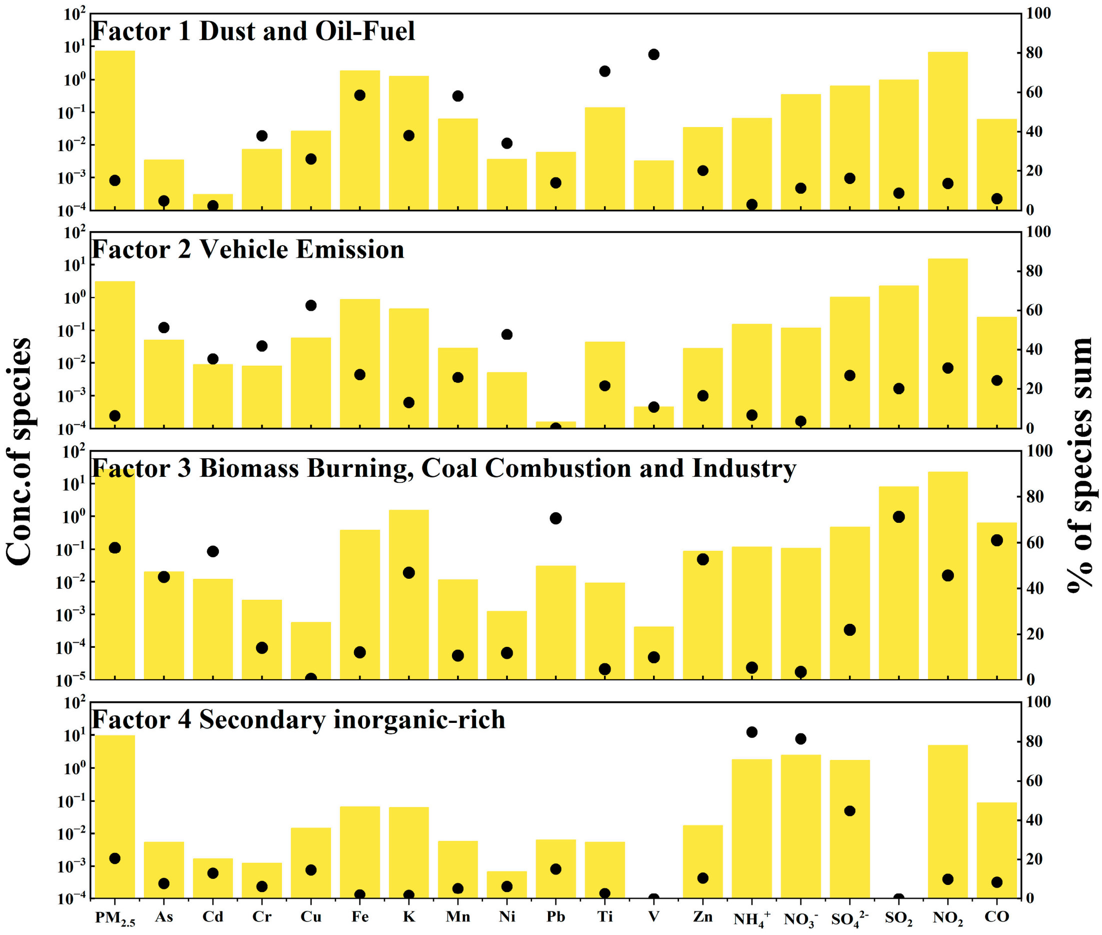

Figure S3. Source profiles of Total Metals.

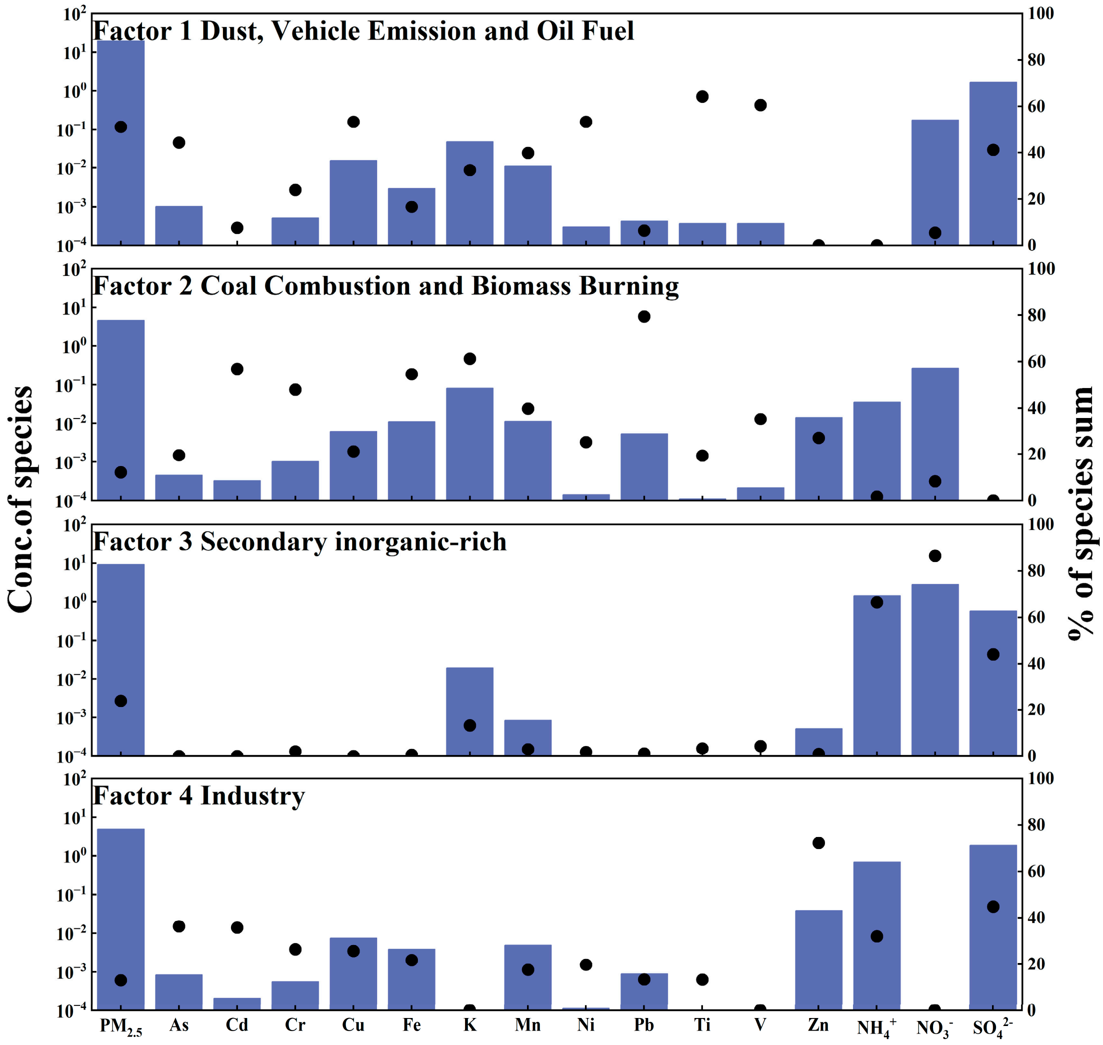

Figure S4. Source profiles of Water-soluble Metals.
